# Kidney Cells Regeneration: Dedifferentiation of Tubular Epithelium, Resident Stem Cells and Possible Niches for Renal Progenitors

**DOI:** 10.3390/ijms20246326

**Published:** 2019-12-15

**Authors:** Nadezda V. Andrianova, Marina I. Buyan, Ljubava D. Zorova, Irina B. Pevzner, Vasily A. Popkov, Valentina A. Babenko, Denis N. Silachev, Egor Y. Plotnikov, Dmitry B. Zorov

**Affiliations:** 1 Faculty of Bioengineering and Bioinformatics, Lomonosov Moscow State University, 119992 Moscow, Russia; 2 A.N. Belozersky Institute of Physico-Chemical Biology, Lomonosov Moscow State University, 119992 Moscow, Russia; 3 V.I. Kulakov National Medical Research Center of Obstetrics, Gynecology and Perinatology, 117997 Moscow, Russia; 4 Sechenov First Moscow State Medical University, Institute of Molecular Medicine, 119991 Moscow, Russia

**Keywords:** renal stem cells, differentiation, scattered tubular cells, papilla, niches

## Abstract

A kidney is an organ with relatively low basal cellular regenerative potential. However, renal cells have a pronounced ability to proliferate after injury, which undermines that the kidney cells are able to regenerate under induced conditions. The majority of studies explain yielded regeneration either by the dedifferentiation of the mature tubular epithelium or by the presence of a resident pool of progenitor cells in the kidney tissue. Whether cells responsible for the regeneration of the kidney initially have progenitor properties or if they obtain a “progenitor phenotype” during dedifferentiation after an injury, still stays the open question. The major stumbling block in resolving the issue is the lack of specific methods for distinguishing between dedifferentiated cells and resident progenitor cells. Transgenic animals, single-cell transcriptomics, and other recent approaches could be powerful tools to solve this problem. This review examines the main mechanisms of kidney regeneration: dedifferentiation of epithelial cells and activation of progenitor cells with special attention to potential niches of kidney progenitor cells. We attempted to give a detailed description of the most controversial topics in this field and ways to resolve these issues.

## 1. Introduction

Despite the fact that the kidney has relatively low basal cellular regenerative potential, tubular epithelial cells have a pronounced ability to proliferate after injury [1]. However, the complexity of the renal tissue in mammals and the low rate of cell renewal makes it difficult to study kidney regeneration mechanisms. In this regard, there is still no consensus on what cells are responsible for the recovery of tubular epithelium after injury [2]. A number of hypotheses have been proposed about the nature of regenerative potential in the kidney tissue. The majority of studies assign the basis of such regenerative potential either to the dedifferentiation of the mature tubular epithelium or to the presence of a resident pool of progenitor cells in the kidney tissue [3,4].

The hypothesis of dedifferentiation as a mechanism of renal tissue restoration was based on the analysis of proliferation after ischemia/reperfusion (I/R) or exposure to damaging agents showing that more than half of all tubular epithelium becomes positively stained for proliferation markers (PCNA, Ki-67, BrdU) [5,6,7,8]. In addition, some morphological changes were observed in the tubular epithelial cells, which together with the aforementioned data was interpreted as dedifferentiation of these cells [9]. Furthermore, cells indicated the appearance of markers of an embryonic kidney, which could be assumed as a return to a less differentiated state [10,11,12]. Since then, a lot of evidence has been accumulated about the dominant role of dedifferentiation in the restoration of renal tissue after injury, including data obtained in transgenic animals.

Subsequently, there was additional evidence indicating the possible existence of a population of progenitor cells (so-called scattered tubular cells, STCs) in the adult kidney which had a more pronounced regenerative potential than differentiated tubular epithelium [13,14,15]. These cells were initially found in the kidneys of rodents [13] and then they were also described in humans [16,17]. Human kidneys have become a very convenient object for progenitor cells studying due to the presence of specific marker CD133 with glycosylated epitope being a “gold standard” to consider these cells as progenitor cells in humans [16,18], as well as in some other mammals [19,20]. Lack of this marker in rodents forces to use other markers for identification of the progenitor population there and determines the need for experiments with transgenic animals expressing fluorescent markers in progenitor cells [21]. A large number of such markers have been proposed (Table 1 and Table 2), which apparently characterize the population of progenitor cells in both human and rodent kidneys [22,23,24].

The identification of cells responsible for the restoration of tubular epithelium is in the scope of regenerative medicine [66,67]. This review examines the main mechanisms of kidney regeneration: dedifferentiation of the epithelium and activation of progenitor cells with special attention to potential niches of kidney progenitor cells. We attempted to give a detailed description of the most controversial issues in this area. In particular, we considered issues based on defects of techniques involved in the detection of progenitor cells and on the inability of discrimination of tubular epithelium proliferation from progenitor cells preexistence.

## 2. Dedifferentiation or Recruitment of Progenitor Cells?

### 2.1. Dedifferentiation

In the kidneys of adult organisms, a renewal rate the cell population is very slow, however, it dramatically enhances after injury [5]. Staining for various proliferative markers, for example, proliferating cell nuclear antigen (PCNA), Ki-67, and evaluating the accumulation of probes such as bromodeoxyuridine (BrdU) showed that injury-induced cell proliferation in the kidney tissue is not associated with some specific regeneration centers, but goes stochastically [7,8]. In this regard, the first hypothesis explaining the restoration of lost renal cells was the dedifferentiation of the tubular epithelium [5,68]. For a long time, it was believed that any renal epithelial cell has a regenerative potency in response to injury [9,69,70].

After exposure to a damaging factor, a peak of proliferation in the kidney tissue was observed usually occurring on the 2nd day, whereas normal epithelial morphology is normally restored within 5–7 days after challenge [1]. Histological analysis of the kidney tissue distinguishes 4 stages of the regeneration process. At the first stage, the death of tubular epithelium is observed, occurring by apoptosis, necrosis, or another death mode, and it is usually accompanied by an inflammatory reaction. In the second stage, survived tubular cells exhibit changes in normal differentiated epithelial phenotypes, such as a loss of brush border, tubular flattening, and rapid loss of cell polarity [71,72]. During this stage, cells undergo epithelial-mesenchymal transition, detected by overexpression of vimentin, which is a marker of mesenchymal cells [9,52]. The third phase is associated with increased levels of growth factors, such as IGF1, HGF, FGFs, and enhanced proliferation of a majority of kidney cells [73]. Growth factors stimulate cells in the G_0_ phase and promote their entry into the cell cycle [74]. The regeneration process is terminated after the recovery of the normal morphology of epithelial cells and restoration of nephron function [75]. Thus, regeneration through dedifferentiation refers to the sequence of histological changes including loss of mature epithelium morphology, epithelial-mesenchymal transition, proliferation to replace lost cells, and re-differentiation [5].

S3 segment of the proximal tubule located near the cortico-medullary junction is known as the most vulnerable part of the nephron [76,77]. Remarkably, the S3 segment also exhibits the most pronounced proliferation after injury compared to other segments of the nephron [78]. Therefore, the majority of studies investigating mechanisms of kidney regeneration are focused on this particular area. Double staining with chlorodeoxyuridine (CldU) and antibodies against Ki-67 revealed that within 48 h after I/R more than 55% of the cells, mainly in the S3 segment, reentered the cell cycle or even passed the S phase [8].

There is a direct histological confirmation of the dedifferentiation of tubular epithelium. In the injured tubules, dividing cells were detected revealing both epithelial and proliferative markers [52,69]. Particularly, dividing cells in the S3 segment of proximal tubules and in the distal tubules had a basolateral expression of Na-K-ATPase (a marker of terminal epithelial differentiation) at the same level as neighboring non-proliferating cells [69], and cells survived after injury carrying intact nuclei actively proliferated and expressed vimentin. Paradoxically, actively proliferating cells continued the expression of Kim-1 [79], a well-known marker of the injured proximal tubular epithelium [80]. Usually, around 35–50% of survived kidney cells begin to express this protein in response to injury [81]. In a strange way, the co-expression of an alarming damaging factor Kim-1 with proliferative factor vimentin in tubular cells after an injury has been currently interpreted as evidence for the proliferation of injured epithelium [8]. It is unclear, whether it reflects the compensatory mechanism for replenishment renal loss of functionality, although it seems dangerous for the organism to reproduce damaged cells.

In addition to vimentin, during kidney regeneration markers specific to kidney development appeared, i.g., Pax-2, and neural cell adhesion molecule 1 (NCAM1). Transcription factor Pax-2 is almost not expressed in adult kidneys, except the collecting ducts and papilla [11]. However, after ischemic or nephrotoxic kidney injury, Pax-2 expression is significantly increased in the survived tubular epithelium, indicating the appearance of cells with immature phenotype [11,49]. NCAM1 is widely represented during nephrogenesis, but it is not detected in the differentiated tubular epithelium [49]. However, upon injury or isolation of kidney cells for culturing, epithelium starts to express NCAM1 again [37]. NCAM1-positive cells exhibit features of epithelial-mesenchymal transformation and possess robust clonal capacity, adopting a progenitor phenotype [82].

Similarly to Pax-2 and NCAM1, another marker of dedifferentiation, Sox9, is actively involved in embryogenesis [51], but is not presented in the kidney tissue of adult organisms [83]. Sox9 expression increases by more than 20-fold 24 h after I/R and its elevated level persists up to 30 days after injury [51]. Over 40% of Sox9-positive cells also express Ki-67 and locate in a scattered-like manner, mainly in the proximal tubules. Sox9+ cells co-express injury markers, neutrophil gelatinase-associated lipocalin (NGAL) and Kim-1, which may indicate that these cells represent injured epithelium. In addition, experiments were performed using lineage tracing showed that Sox9+ cells really contributed to kidney regeneration [51].

In addition, nestin, the protein belonging to intermediate filaments, was recently proposed as a marker of dedifferentiation. After subtotal nephrectomy, the expression of nestin was increased in epithelial cells bordering the injured area [45]. These cells actively proliferated, so the expression of nestin was suggested as a dedifferentiation-associated feature.

### 2.2. Progenitor Cells

#### 2.2.1. Progenitor Cells in Rodent Kidneys

The first assumption of the presence of progenitor cells in the kidneys arose in the study of Maeshima et al. [13]. In this study, adult intact rats were treated with BrdU, which accumulated in cells in the S-phase [84]. Analysis of kidney cells was conducted 2 weeks after the end of the 7-days BrdU administration and allowed to identify cells with the slow cell cycle. These cells were scattered among other cells of the proximal and distal tubules, so they later became known as scattered tubular cells (STCs), or label-retaining cells (LRCs). To detect possible progenitor properties of LRCs, rats were exposed to I/R, and it was revealed that the number of BrdU+ cells significantly increased 24 h after I/R, most of them were located in 2-cell clusters and expressed PCNA. These cells expressed vimentin as well, and at day 10 began to express E-cadherin (a marker of differentiated epithelium) [85]. Similar data on the presence of LRCs were obtained in newborn mice in which BrdU+ cells were located mainly in the S3 segment and in the papilla [28].

In addition to label retention, these presumably progenitor cells have a more pronounced regenerative potential than non-LRCs. For example, on a three-dimensional gel substrate, they formed tubule-like or tubulocystic structures in response to growth factors treatment [26]. When transplanted into the metanephric kidney, these cells were embedded into epithelial components of a nephron, including proximal tubules, where they demonstrated 3.5–13 times higher proliferative potential [15]. Cells isolated from the S3 segment of adult rat kidneys were able to reconstruct a three-dimensional kidney-like structure in vitro, having all parts of the nephron, including the glomerulus, tubules, and collecting ducts [35]. Moreover, S3-segment cells injected into adult kidney right after ischemia were found in the cortex and medulla confirming their participation in regeneration [14]. However, despite implantation into the kidney tissue, these cells did not cause any significant physiological effects on kidney function estimated by serum creatinine and urea.

A comparative analysis of human and rat renal progenitor cells revealed a population of human scattered tubular cells with a small amount of cytoplasm and mitochondria, without a brush border, which was positive for CD24, CD133 and other progenitors markers [16]. No similar cells with atypical morphology were found in intact rats. The study of renal progenitor cells in rats is complicated by the lack of specific expression of CD24 and glycosylated form of CD133, therefore the search was carried out by the staining for vimentin and CD44 which is another marker for stemness. While absent in intact tissue, vimentin-positive cells with atypical morphology appeared de novo after unilateral ureteral obstruction. The cells (appeared in areas with severe tubules damage) were located singly or in chains of cells and did not have a brush border [16]. However, the emergence of progenitor cells de novo may be only the result of the dedifferentiation.

A similar situation was observed for transcription factor Sox9, which sometimes is used as a marker of progenitor STCs in mice [50]. For a number of tissues, Sox9 is considered to characterize the population of progenitor cells, for example, in hair follicles, retina, and nerve tissue [12,86,87]. However, in renal tissue, cells begin to actively express Sox9 only after injury. Therefore, although they possess many features of progenitor cells (expression of CD133 and Lgr4, the ability to differentiate into adipogenic, osteogenic and chondrogenic cultures), their appearance can be attributed only to dedifferentiation of some renal cells [50].

Sall1, CD24, Sca-1, and nestin have also been proposed as markers of renal progenitor cells. Sall1 is a transcription factor involved in nephrogenesis [88]. Analysis of its expression in the adult kidney revealed that about 0.5% of all cells contained Sall1 located mainly in the cortico-medullary junction [39]. After I/R, 90% of Sall1-positive cells started to proliferate and 5% of these cells showed asymmetric cell division with one of the two adjacent Sall1-positive cells. CD24 is a glycoprotein that is selectively expressed in immature cells of different tissues and it is almost absent in differentiated cells [89]. The presence of this marker was shown in the population of progenitor cells in rodent kidneys [15], however, it is not always possible to obtain its specific staining [16]. Another important marker is Sca-1, which was initially detected as a marker of hematopoietic stem cells until its association with renal progenitor cells was shown [14,15,36,40]. Finally, the aforementioned nestin, intermediate filaments protein, unambiguously associated with progenitor cells in nervous tissue [90], was also found in the cells of some kidney compartments, which are considered as niches for progenitor cells, particularly, the papilla and cortico-medullary junction [62].

In a recent study, the analysis of kidney progenitor cells was performed using transgenic mice with doxycycline-induced random labeling of all tubular epithelial cells by permanent recombination of a single-color-encoding gene [46]. Analysis carried out 30 days after an acute kidney injury (AKI) showed that tubules consisted of clones of cells with the same color and mainly located in the S3 segment of the kidney. Calculations based on the percentage of differently colored clones demonstrated that only a small number of epithelial cells underwent mitosis after I/R, most of them were Pax-2-positive. During regeneration, these cells formed single-colored clones of more than 10 cells. Only Pax-2+ cells fully passed the mitotic cycle, whereas the rest of the tubular epithelium has undergone an endoreplication cycle [46].

Further evidence for the presence of progenitor cells pool in rodent kidneys came from the study of Rinchevich et al. using the so-called rainbow mice [91]. These mice express multicolored reporter constructs allowing to detect cells with segment-specific clonogenic and proliferative potential. One month after the induction of reporter protein expression in intact mice, the clones were observed as small groups of 2–3 cells with the same color. After a longer period, the clones increased to groups consisting of more than 8 cells and they were located both in the cortical substance and in the medulla, in particular, the papilla. The findings showed that tubulogenesis exists in the adult kidney and only a subset of adult epithelial cells was responsible for it. The number of clones of the same color increased after I/R, and most of them (60%) were found in the cortical substance. Thus, this study proved the presence of a functional population of renal progenitor cells [91]. However, it still remains unclear whether these cells belong to a separate pool or they originate from the epithelium transiently acquiring a progenitor phenotype [92].

#### 2.2.2. Progenitor Cells in Human Kidneys

After the discovery of progenitor cells in rodent kidneys, there were studies demonstrating the existence of such cells in human kidneys [27,32]. A population of cells with morphology and progenitor properties different from normal epithelial cells was isolated in the proximal tubules. The main markers of this population were CD24, CD133, and vimentin, and cells were scattered throughout the proximal tubule in the normal human kidney [16]. If compared to conventional epithelial cells, these cells contained less cytoplasm, fewer mitochondria, and had no brush border [16]. The average number of progenitor cells in the cortical substance of the human kidney was estimated at 0.5–4% [17,32] or slightly more (3%–12%) [31]. Most CD133+ cells in the human kidney are located in the S3 segment of the proximal tubules [27,93]. It is noteworthy that this region is most susceptible to damaging factors, but at the same time, it has a remarkable capacity to rerestore its structure and function [77,94].

A convenient feature of human kidney progenitor cells, absent in similar rodent cells, is the presence of CD133, a specific marker of undifferentiated cells. Although CD133 is abundant in both immature and differentiated cells, specific glycosylated epitopes (CD133/1 and CD133/2) have been found only on immature cells in humans [95,96], such as hematopoietic stem/progenitor cells and tissue-specific progenitor cells [97]. The glycosylated form of CD133 has been shown to be expressed in S-shaped bodies in the fetal kidney and co-expressed with Ki-67 [93]. Thus, CD133 is a widely used marker of progenitor cells, however, when staining for this antigen, it is very important to monitor the specificity of antibodies, to exclusively recognize the epitope related to undifferentiated cells only [98]. For confirmation of the results of CD133 detection, cells often are examined for CD24, which usually co-expresses with CD133 [99].

It has been shown that cells positive for CD24/CD133 in various parts of the nephron can be considered as a population of resident progenitor cells. They have the ability to expansion, self-renewal, and epithelial differentiation both in vitro and in vivo [16,17,31,32]. In culture, they are able to differentiate into tubular, osteogenic, neuronal, adipose cells and to repair tubular structures [100]. In vitro, they have the ability to form spheres, which is a specific feature of stem cells [18] and to proliferate for a long time without signs of cell senescence [25]. These cells contain fewer mitochondria than conventional epithelial cells [16], which was confirmed by electron microscopy using gold-conjugated vimentin antibodies, as well as by double immunofluorescence staining for CD133 and mitochondrial markers [31]. However, despite the reduced mitochondrial content, CD133+ cells demonstrate increased Bcl-2 expression [16,18]. CD133 itself is known to participate in glucose uptake [101], and stem cells, in general, are prone to anaerobic metabolism [102]. Probably, the combination of these factors explains the increased resistance of these cells to apoptosis [17].

In addition, cells expressing CD24 and CD133 have a pronounced regenerative potential when administered to mice with severe combined immunodeficiency (SCID) exposed to I/R [100]. A population of human CD133+ papillary cells also possesses a profound nephroprotective potential when administered to rats subjected to glycerol-induced acute tubular damage. It provides restoration of kidney function, preventing tubular necrosis and stimulating proliferation of their own resident cells [30]. CD133+ cells also show signs of proliferation in the renal biopsy material from patients suffering renal insults [17,31]. Despite the fact that the high proliferative activity of putative progenitor cells has been widely shown, it should be kept in mind that cells can behave in vitro in a completely different way than in the organism [48]. For example, human CD133+ cells injected after kidney injury have been shown to be implanted into the tubules of embryonic kidneys, but not in adult rat kidneys [103].

It was found that, apart from CD24 and CD133, another 49 proteins were expressed in the kidney in the same scattered pattern [16]. Among them, there are already mentioned Pax-2 and Sox9, however, colocalization with CD24 or CD133 was shown only for vimentin, S100A6 and several other proteins, e.g., aldehyde dehydrogenase 1 [18]. Recently, a transcriptional profile of CD133+ cells was obtained by RNA sequencing [25]. Overexpression of CD24, PAX-2, vimentin, aldehyde dehydrogenase 1, S100A6, as well as of some other markers were detected.

The existence of progenitor cells distributed in the kidney in a scattered-like manner raises the question of their origin in the process of nephrogenesis [17]. CD133 and CD24 are expressed under kidney development, with the main cluster located in the urinal pole of Bowman’s capsule, and a small portion located in the distal tubules in the junction with the glomerulus. It is assumed that during the growth of the kidney, the cells spread and formed the STCs observed in the adult kidney [104]. This once again proves the indissoluble connection of STCs of tubules with the population of glomerular parietal cells, which are recognized as a pool of progenitor cells for podocytes and contain the same markers as STCs [105].

### 2.3. State of the Art

Thus, there is still a discussion about the genuine nature of the regenerating mechanisms in the adult kidneys of humans and other mammals [1]. The main problem is the lack of specific methods and unique markers for distinguishing between dedifferentiation and progenitor cells’ preexistence [2]. For instance, vimentin, which in some studies used as a marker of dedifferentiation and epithelial-mesenchymal transition [9], is also overexpressed in the population of cells defined as progenitors [5].

A similar discussion is going around Kim-1 [106], which is a common marker of the injured proximal tubular epithelium [79,80]. For a long time, the coexpression of vimentin and Kim-1 in the same cells was considered as strict evidence of the dedifferentiation of the injured epithelium as a regenerative mechanism [79]. However, several studies showed that progenitor cells also express Kim-1 after injury [16,18]. To resolve the issue, transgenic mice were created expressing a fluorescent construct under the Kim-1 promoter [42]. The study revealed that Kim-1 was not expressed in renal cells of intact animals. Therefore Kim-1 could not be a marker of the resident progenitor cells. After I/R, in the kidney tissue, clones of cells were found expressing Kim-1, vimentin, Sox9, and Ki67, that was interpreted as a return to the dedifferentiated state rather than a proliferation of resident tubular progenitors. In addition, in this study transcription factor, Foxm1 was described as a new potential marker of dedifferentiated kidney cells [42]. Foxm1 was overexpressed in the injured proximal tubular epithelium, especially in the S3 segment.

The identification of embryonic kidney markers does not resolve the existing contradictions. On the one hand, markers that take part in the process of nephrogenesis should presumably appear during dedifferentiation [1]. On the other hand, a population of progenitor cells, if exists, may also express neonatal kidney markers [23]. For instance, Pax-2 overexpression has been suggested as an argument in favor of the dedifferentiation of mature tubular epithelium after injury [11]. However, in the intact kidney, a population of Pax-2+ cells was also found, which constituted about 10% of cells in the S3 segment [46].

A similar situation occurs around Sox9 [1], initially proposed as a marker of dedifferentiated epithelium due to its expression during nephrogenesis [51]. After an injury, Sox9 colocalized with markers of injured tubular epithelium, such as NGAL and Kim-1 [51]. However, in the intact adult kidney, Sox9-positive cells were found representing a small population of scattered cells that started to proliferate after injury [50], suggesting Sox9 more likely associated with progenitors.

It still remains unclear whether the population of progenitor cells differs from mature tubular epithelium by the number of mitochondria. On the one hand, in adult rat kidney, STCs were characterized by a large number of mitochondria [107]. On the other, in human kidneys, it was found that STCs had a small amount of these organelles [16,31]. Since the content of mitochondria has a very strong effect on cell metabolism, accurate information about the number of these organelles in progenitor cells could help in the development of methods for affecting these cells.

There is a serious limitation in studying renal progenitor cells due to using CD133 as a key marker of undifferentiated cells in human kidneys [108]. Firstly, the glycosylated epitope of CD133 is present in the kidneys of humans, primates, and pigs, but it is absent in rodents [109], which are the main experimental animals. Secondly, the level of glycosylation depends on the stage of cell differentiation [98]. Therefore, the usage of antibodies recognizing CD133 outside the glycosylated epitope can lead to incorrect results [96]. So it is crucial to monitor the specificity of antibodies to the glycosylated epitope in order to selectively determine the pool of progenitor cells. Finally, CD133 antigenic specificity may not only be a limitation of the technique but also indicates differences in the mechanism for kidney regeneration in humans and rodents [2]. For instance, it has been suggested that in humans, progenitor cells could preexist in the tubules, while, in rodents, dedifferentiation might predominate as the main regeneration mechanism [16]. However, this hypothesis was questioned by the detection of progenitor cells in rodent kidneys using other markers [38,50,64].

**Figure 1:**
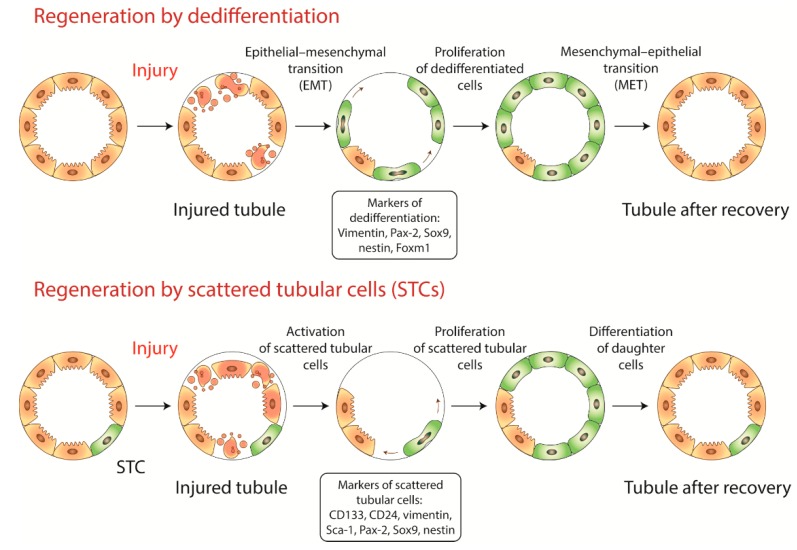
Two major putative mechanisms of kidney tissue regeneration: dedifferentiation of tubular epithelial cells and proliferation of resident renal progenitors with subsequent differentiation.

Thus, the majority of studies support the idea that after injury, the adult kidneys acquire a population of cells with pronounced regenerative potential. However, it remains unclear whether these cells arose from dedifferentiated epithelial cells or from the preexisting population of progenitor cells. The current views on these mechanisms are summarized in Figure 1.

## 3. Renal Papilla as a Niche for Progenitor Cells

Morphologically, papilla belongs to the inner layer of renal medulla and plays a crucial role in urine concentration due to residing Henle’s loop of juxtamedullary nephrons [110]. Some studies suggest papilla as a putative niche for progenitor cells [55,62,111]. This hypothesis is based on the presence of a large number of cells with the slow cell cycle in the papilla and those cells carrying markers of progenitor cells [2]. Moreover, the papilla is a place with unique conditions that are simultaneously hyperosmotic and hypoxic [112]. The hypoxic microenvironment is a distinguishing feature for stem cell niches in the other organs, such as bone marrow and brain [113]. Papilla cells along with STCs in proximal tubules express progenitor cell markers, for instance, glycated CD133 in human kidneys or nestin in rodent kidneys, and these cells change their properties during tissue regeneration [30,61,62]. Furthermore, papilla cells are positive for embryonic kidney markers, for instance, Pax-2 [11] and TNFRSF19 [64], even in intact adult kidneys.

Papilla as a niche for progenitor cells was suggested in 2004 by Oliver et al., who tried to discover renal resident progenitor cells and outline their properties [55]. The research was based on the observation that organ-specific adult stem cells in a number of tissues have a slow cell cycle that can be detected by retention of BrdU, which integrates into DNA molecule during replication [84]. The study was performed on neonatal rats and mice, which are characterized by the ongoing process of nephrogenesis for a few days after birth. Newborn rodents were injected with BrdU solution, and label retention was estimated 2 months later in the kidney tissue. As a result, in papilla, a population of LRCs was found, with a slow cell cycle, which resided mostly in interstitium although some of them were colocalized with markers of tubular epithelial cells. These LRCs were not bone marrow-derived or belong to endothelial cells. However, after I/R, BrdU-positive cells were absent in the cortex and medulla, which refuted the hypothesis about LRCs migration towards injured areas of the kidney [55].

However, the BrdU labeling assay has several restrictions. The assay mechanism bases on the ability of bromodeoxyuridine to replace thymidine during replication with such replacements being detected by specific antibodies [84]. Label levels slowly decrease in the daughter cells when cells divide after label withdraw. Due to the slow cycle, stem and progenitor cells contain the label for a longer time [114]. However, all cells in S-phase accumulate BrdU during its administration that is the main limitation of the assay [7].

As a result of limitations with BrdU labeling assay, there were attempts to detect progenitor cells in the papilla using lineage tracing in transgenic mice expressing green fluorescent protein (GFP)-fused histone protein (H2B-GFP) under tetracycline-sensitive promotor [115]. The assay was based on the high stability of H2B-GFP protein in the cells with a slow cycle. Consequently, stimulation of its expression before the mice’s birth resulted in the detection of cells with a slow cycle even within months after birth [58]. This assay confirmed that cells with slow cycles were located mainly in the papilla, but not in the outer medulla or cortex. Moreover, GFP-positive cells migrated toward the upper part of the papilla where these cells formed chain-like structures of proliferating cells positive for Ki-67 [58].

The population of papilla stem cells was also found in transgenic mice expressing GFP under the nestin promoter [90]. Nestin is considered to be a marker of progenitor cells, including the kidney [60,116]. Those mice had GFP-positive cells mainly in the papilla, and only a small amount was located in the cortico-medullary junction [62]. In the study, evidence was found that GFP-positive cells migrate from the papilla to cortex [90]. The main limitation of the model was a constitutive nestin expression in the adult podocytes and in some endotheliocytes [117,118]. Furthermore, nestin expression in podocytes has been shown to rise during some pathological conditions [119,120].

One more approach for detecting cells with a slow cell cycle is in using mTert-GFP as a reporter system, thus labeling telomerase-expressing embryonic stem cells [59]. On the one hand, such a reporter was chosen because telomerase is a biomarker of stem cells. On the other hand, knockout of mTert leads to the increased severity of AKI, which is believed to be associated with inhibiting of mTert-expressing renal progenitor cell population [121]. The majority of GFP+ cells were observed in the papilla (about 10% of all papillary cells); a small amount was detected in the outer medulla, but not in the cortex. Colocalization with the other cell type-specific proteins showed that mTert was expressed primarily in epithelial cells [59].

Based on the suggestion that papillary progenitor cells have the same cell markers as other tissues progenitor cells GFP-positive cells from H2B-GFP transgenic mice were obtained by fluorescence-activated cell sorting (FACS), and their specific markers were defined. Only protrudin (Zfyve27) demonstrated selective expression in the papilla and it was absent in the other kidney areas [65]. Protrudin-positive cells appeared not to contribute to normal kidney maintenance, however, after severe kidney injury, cells started to proliferate and generate long tubular segments located preferentially in the kidney medulla [65]. Additionally, these cells had many morphological characteristics specific to migratory cells [122].

**Figure 2:**
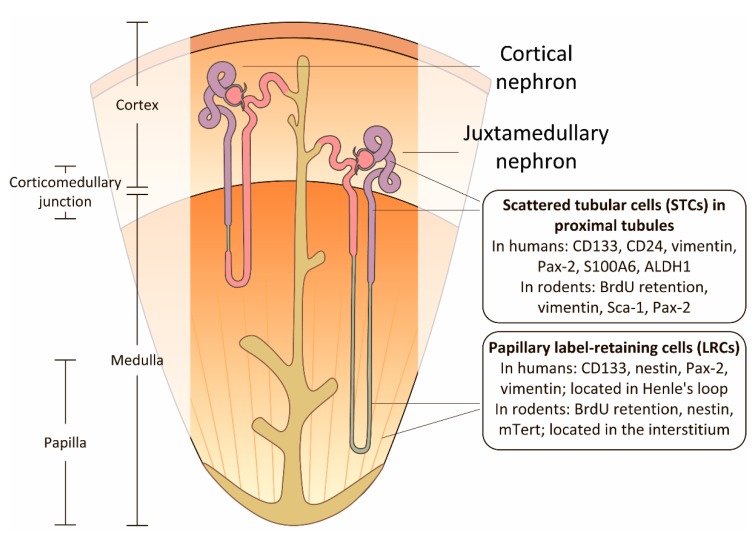
Major suggested niches of renal progenitor cells based on the immunophenotyping with specific surface markers and label retention approach. There are two putative niches for progenitor cells: proximal tubules (especially their S3-segments) and papilla. Progenitor cells in human and rodents kidneys are characterized by slightly different markers listed in Table 2. It is worth noting some differences in the location of progenitor cells: in the papilla of rodent kidney, label-retaining cells (LRCs) reside in the interstitium, while in human kidneys these progenitors constitute the Henle’s loop among differentiated cells of the nephron.

Considering this data, it was suggested that different kidney areas might have different progenitor cell pools [65] (Figure 2). For instance, papillary LRCs could be activated only in response to severe injury and they restore mainly epithelium in the medulla. This suggestion correlates with the experiments performed on the other epithelial tissues which showed the existence of progenitor cell pools responding to damaging factors being responsible for restoring anatomically various parts of an organ [123,124].

Interestingly, papillary progenitor cells were found both in rodents [55,62] and human kidneys [60,61], but with some differences in the localization of the cells. In rodents, a preferential interstitial localization of progenitor cells was observed [55,58,62], while human progenitors were found primarily inside Henle`s loops [60]. Both in rodents and humans, these cells were colocalized with the conventional progenitor cell markers. It was shown that CD133+ and nestin+ cells in Henle`s loop were located both in the papilla and cortex of the human kidneys. CD133+ cells obtained from the human papilla actively proliferated; after injection into mice embryonic kidney, they integrated into tubules and were involved in tubulogenesis [60]. Similarly, rodent papilla contained cells expressing nestin and telomerase [59,62,125], and papillary cells from pig kidneys were positive for progenitor cells markers CD24 and CD133, and they had myogenic, osteogenic, and adipogenic differentiation potential [111].

To date, the involvement of the papillary cells in kidney regeneration is not fully understood. Whether it is achieved through progenitors migration and integration into tubules, or through paracrine mechanisms is not clear. Hypothesis about proliferation and migration of progenitors daughter cells are based on almost 9 fold decrease of LRCs in papilla 3 weeks after I/R injury [55]. However, in GFP-nestin mice such a decrease in LRCs was not shown after I/R [62]. The question is: why so many papillary LRCs lose BrdU label after injury, whereas only a fraction of them proliferate after injury, and apoptosis is not observed in this area [114].

The migration of papillary LRCs was confirmed in the single study using GFP-nestin mice when papillary nestin-GFP+ cells migrated to cortex and medulla after I/R [62]. Other studies with more evidence-based data demonstrated that migration is limited by the medulla [58,65]. Moreover, the mTert-GFP mouse model showed no evidence of the migration of mTert LRCs from the papilla in response to injury [59]. Humphreys et al. reiterated the study with BrdU administration during nephrogenesis; LRCs in their experiments neither migrated during repair from I/R nor selectively proliferated in those conditions [8]. Furthermore, Ki-67 staining in kidneys of mice injected with CldU during infancy showed that LRCs did not demonstrate proliferation after injury in the cortex and medulla [8]. “Chains” of proliferating Ki-67+ cells found in upper papilla did not colocalize with CldU-positive cells. However, despite the negative results with LRCs, Humphreys et al. did not refute that papilla cells might affect other cells via the paracrine mechanisms [54].

Thus, various methods indicated that kidney papilla contains a cell population with a slow cell cycle involved in regeneration processes in the other parts of the kidney [8,28,55]. However, the biological significance of the long-term BrdU-retaining population is not fully understood yet. These cells could be a population that differentiated in the kidney as early as during embryogenesis and then have never proliferated for any reason. On the other hand, LRCs rapidly exit the cell cycle and undergo much fewer divisions than tubular epithelium thus having more significant regenerative capacity after injury [56]. Due to a large number of contradictions in this area, it is difficult to accept unambiguously that a kidney papilla is a niche of progenitor cells. Further experiments are required to clarify the biological significance of this renal papillary cell population and to identify possible mechanisms of its role in regeneration.

## 4. Potential Approaches Affecting Kidney Regeneration

A discussion around the presence of progenitor cells in the kidneys of adult organisms appears from the requests of regenerative medicine, because if such cells exist, it would be possible to develop approaches selectively enhancing kidney regeneration [66]. The development of such approaches is possible in the case of dedifferentiation as the main mechanism of regeneration, as well. However, the presence of a pool of progenitors with specific markers and their own physiological characteristics increases the chances to find a successful strategy. Therefore, numerous studies are focused on searching and phenotyping these cells [67].

One of the cell therapy approaches is the use of resident progenitor cells obtained from the kidney by isolation, cultivation and subsequent transplantation (autologous or allogeneic) in the injured organ. Such design is frequently described in the experimental works performed on rodents. Recent studies showed that cells could integrate into the tubules of neonatal and adult kidneys, and then either directly or indirectly could influence the regeneration of renal tissue through the paracrine mechanisms [15,16,17,35]. However, not in all studies the real improvement of the organ functions was achieved [33]. It is known that cell therapy with resident kidney progenitor cells reduces the activation of apoptosis and inflammation [126], improves angiogenesis [127], reduces fibrosis [128,129], and even increases animal survival after kidney injury [130].

On the other hand, attempts are continuing to develop approaches for affecting resident progenitor cells, for example, to increase the activity of glomerular parietal cells, which are known to be progenitors of podocytes. Some compounds, such as glycogen synthase kinases 3-α and -β (GSK3s) inhibitor 6-bromoindirubin-3-oxime (BIO) [131], notch signaling inhibitors [132], interferon [133], steroids [134], and some others enhanced the proliferation of parietal cells and mediated their differentiation into podocytes in vitro [135]. Perhaps, compounds exist that would selectively affect STC or other possible pools of progenitor cells.

However, it should be taken into account that excessive activation of kidney progenitor cells could have unwanted side effects on organ function. For instance, the above-mentioned activation of parietal cells is observed in glomerulonephritis and diabetic nephropathy and does not lead to a positive outcome. Excessive proliferation can generate lesions of cells, extracapillary crescentic glomerulonephritis, collapsing glomerulopathy, tip lesions, and ultimately these processes compromise the normal functioning of the glomerulus [136].

## 5. Summary

Obviously, a kidney has a pronounced regenerative potential, however, its cellular basis is still not fully understood. No doubt that some renal cells are responsible for the regeneration of the kidney, but whether these cells initially have progenitor properties or they obtain a “progenitor phenotype” during dedifferentiation after an injury, still stays the main question. The major stumbling block in resolving the issue is the lack of specific methods for distinguishing between dedifferentiated cells and resident progenitor cells [2]. The complexity of the morphological structure of the kidney and the evidence of the existence of populations of different progenitor cells led to the suggestion that different parts of the kidney may have various progenitor cell pools. Another hypothesis is that diverse cell populations are activated in response to different damaging stimuli [137]. Finally, it is possible that two mechanisms of regeneration may coexist in the kidney, complement and compensate each other [2,92].

Transgenic animals are a powerful tool to solve this problem, as well as new methods, for example, single-cell transcriptomics, which has already been successfully used to study the kidney recovery after injury [43,138]. It is hoped that the application of these approaches will soon lead to the discovery of the true source of regenerative potential in the kidney and allow regenerative medicine to choose targeted methods for renal tissue regeneration after injury.

## Figures and Tables

**Table 1:** Conventional markers used for the detection of progenitor cells or the dedifferentiation of tubular epithelial cells. Markers, which are used for progenitor cells detection, are partially different for human and rodent kidneys. Foxm1 is the only marker specific for dedifferentiation. Other markers are used both for dedifferentiated cells and progenitor cells and not selective. Empty fields indicate that the marker was not reported for specified conditions.

	Marker	Progenitor Cells		Dedifferentiation
		*Human*	*Rodents*	
Markers of progenitor cells	ALDH1	[18,25]	-	-
	BrdU retention	Not applicable	[13,26,27,28]	-
	CD24	[16,17,18,25,29,30,31]	[15]	-
	CD44	[30,32]	[33]	-
	CD73	[30,32]	-	-
	CD133	[16,17,18,29,30,31,32,34]	Not applicable	-
	C-kit	-	[14,35]	-
	Musculin	-	[36]	-
	NCAM1	[37]	-	-
	NFATc1	-	[38]	-
	S100A6	[16,18,25]	-	-
	Sall1	[25,37]	[39]	-
	Sca-1	-	[14,15,35,36,40]	-
	SIX2	[37,41]	-	-
Marker of dedifferentiation	Foxm1	-	-	[42,43]
Non-selective markers	Nestin	[44]	[35]	[45]
	Pax-2	[25,30,32,34,37,44]	[14,33,35,46]	[8,11,47,48,49]
	Sox9	-	[50]	[42,51]
	Vimentin	[16,17,18,25,30,31,44]	[13,14,26,33,35]	[9,42,47,48,52,53]

**Table 2:** Markers of progenitor cells located in the papilla of human or rodent kidney.

Marker	The Papilla of Human Kidney	The Papilla of Rodent Kidney
BrdU retention	Not applicable	[27,54,55,56,57,58,59]
CD133	[60,61]	Not applicable
mTert	-	[59]
Nestin	[60,61]	[55,62]
Oct4	[60,61]	-
Pax-2	[61]	-
Sca-1	-	[63]
Troy/TNFRSF19	-	[64]
Vimentin	[61]	-
Zfyve27	-	[65]

## Abbreviations

I/R	Ischemia/reperfusion
AKI	Acute kidney injury
STCs	Scattered tubular cells
LRCs	Label-retaining cells
